# The unseen impact of subclinical hypothyroidism on lipid profile and cardiovascular risk

**DOI:** 10.1016/j.bbrep.2026.102605

**Published:** 2026-04-24

**Authors:** Muhanad Salah Mawlood

**Affiliations:** a Clinical Analysis Department, College of Pharmacy, Hawler Medical University, Erbil, Kurdistan Region, Iraq; b Department of Pharmaceutical Technology, Faculty of Pharmacy, Tishk International University, Erbil, Kurdistan Region, Iraq

**Keywords:** Subclinical hypothyroidism, Dyslipidemia, Thyroid-stimulating hormone, LDL/HDL ratio, Lipid metabolism

## Abstract

**Background:**

Subclinical hypothyroidism (SCH), defined by elevated thyroid-stimulating hormone (TSH) with normal circulating thyroid hormones, is a common endocrine disorder that frequently remains clinically silent. Emerging evidence suggests that even mild thyroid dysfunction may influence lipid metabolism and contribute to early cardiovascular risk. However, the extent to which TSH levels reflect lipid abnormalities in SCH remains controversial.

**Objective:**

This study aimed to evaluate the influence of SCH on lipid metabolism and to determine whether TSH levels are associated with alterations in lipid profile parameters and atherogenic cardiovascular risk markers.

**Methods:**

A cross-sectional comparative study was conducted including 40 participants: 20 patients diagnosed with SCH and 20 euthyroid controls matched for demographic characteristics. Serum concentrations of TSH, free triiodothyronine (FT3), and free thyroxine (FT4) were measured alongside lipid profile parameters, including total cholesterol, triglycerides, low-density lipoprotein cholesterol (LDL-C), and high-density lipoprotein cholesterol (HDL-C). The LDL/HDL ratio was calculated as an indicator of atherogenic cardiovascular risk. Independent sample *t*-tests were applied to compare study groups, and linear regression analysis was performed to evaluate associations between TSH levels and lipid parameters.

**Results:**

Individuals with SCH demonstrated significant lipid alterations compared with euthyroid controls. Triglyceride levels were significantly higher (165.7 ± 60.8 vs. 92.9 ± 39.8 mg/dL; *p* = 0.0136), while HDL-C levels were significantly lower (50.8 ± 12.4 vs. 58.6 ± 11.3 mg/dL; *p* = 0.0136). The LDL/HDL ratio was also significantly elevated in the SCH group (2.25 ± 1.05 vs. 1.57 ± 0.56; *p* = 0.0027), indicating a more atherogenic lipid profile. In contrast, total cholesterol and LDL-C showed modest but statistically non-significant increases. Regression analysis revealed weak and non-significant correlations between TSH levels and lipid parameters.

**Conclusion:**

SCH is associated with unfavorable lipid alterations characterized by elevated triglycerides, reduced HDL-C, and an increased LDL/HDL ratio, suggesting early atherogenic risk despite normal thyroid hormone levels. These findings highlight the importance of comprehensive lipid assessment in SCH and suggest that the LDL/HDL ratio may serve as a more sensitive indicator of cardiovascular risk than TSH alone.

## Highlights

• Subclinical hypothyroidism is associated with significant alterations in lipid metabolism.
• Patients with SCH exhibited significantly higher triglycerides and lower HDL-C levels.
• The LDL/HDL ratio was significantly elevated in SCH patients, indicating increased atherogenic risk.
• TSH levels showed weak and non-significant correlations with lipid parameters.
• LDL/HDL ratio may serve as a more sensitive marker of cardiovascular risk than TSH alone.

## 1 Introduction

Subclinical hypothyroidism (SCH) is considered a mild form of thyroid dysfunction when compared with overt hypothyroidism. Overt hypothyroidism is characterized by decreased secretion of thyroid hormones (T3 and T4) accompanied by elevated thyroid-stimulating hormone (TSH) levels. In contrast, SCH is defined by increased serum TSH concentrations while circulating levels of T3 and T4 remain within the normal range [1]. This condition is frequently asymptomatic and often goes undetected unless identified through routine laboratory investigations [2]. Although individuals with SCH generally do not present with typical symptoms of hypothyroidism—such as fatigue, weight gain, and cold intolerance—accumulating evidence suggests that even this subtle thyroid dysfunction may produce metabolic effects, particularly in relation to lipid metabolism [3,4].

The thyroid gland, positioned in the anterior part of the neck, produces two major hormones—triiodothyronine (T3) and thyroxine (T4)—that play a vital role in regulating several metabolic activities, including lipid metabolism, cardiovascular performance, and overall energy balance. [5]. Thyroxine (T4) primarily functions as a precursor hormone, whereas triiodothyronine (T3) represents the biologically active form generated through peripheral conversion of T4 in organs such as the liver and kidneys [6]. Thyroid hormones regulate a wide range of physiological processes, including cholesterol synthesis, lipoprotein metabolism, fatty acid oxidation, and cardiovascular activity. Because these hormones influence almost all tissues in the body, they are considered essential regulators of metabolic homeostasis and lipid balance [7].

Thyroid hormones, particularly triiodothyronine (T3), play a critical role in the regulation of lipid metabolism through multiple molecular mechanisms. One of the primary pathways involves the direct upregulation of low-density lipoprotein receptor (LDLR) gene expression. T3 binds to thyroid hormone response elements (TREs) located on the promoter region of the LDLR gene, thereby enhancing LDLR mRNA transcription and increasing hepatic LDL receptor synthesis. In hypothyroid states, reduced circulating T3 levels lead to diminished transcriptional activation of the LDLR gene, resulting in a substantial decline in LDLR mRNA expression, which may decrease by approximately 50% [8].

In addition to this direct genomic effect, thyroid hormones regulate LDLR expression indirectly through activation of sterol regulatory element-binding protein-2 (SREBP-2), a key transcription factor involved in cholesterol homeostasis. Reduced T3 concentrations decrease the nuclear availability of SREBP-2, thereby suppressing LDLR gene transcription and impairing hepatic clearance of circulating low-density lipoprotein cholesterol (LDL-C). Furthermore, decreased thyroid hormone levels are associated with increased concentrations of proprotein convertase subtilisin/kexin type 9 (PCSK9), a protein that promotes degradation of LDL receptors on the hepatocyte surface. The combined effects of reduced LDLR synthesis and enhanced receptor degradation lead to a marked reduction in hepatic LDL receptor density, resulting in a decreased fractional catabolic rate of LDL particles and subsequent elevation of circulating LDL-C levels [[9], [10], [11]].

A number of studies have reported a relationship between SCH and dyslipidemia, which is commonly characterized by increased levels of LDL-C, total cholesterol, and triglycerides, along with decreased concentrations of high-density lipoprotein cholesterol (HDL-C) [12]. These alterations contribute to the development of an atherogenic lipid profile, thereby increasing the likelihood of coronary heart disease, atherosclerosis, and other cardiovascular conditions [13]. Individuals with hypothyroidism, including those diagnosed with SCH, may therefore face an increased risk of cardiovascular events, heart failure, and cardiovascular mortality [[14], [15], [16]]. Even slight elevations in TSH levels may reduce the activity of LDL receptors, leading to impaired clearance of LDL-C and increased circulating LDL-C concentrations, which represent an established cardiovascular risk factor [17].

Furthermore, SCH may affect the activity of lipoprotein lipase (LPL), an enzyme responsible for the hydrolysis of triglycerides present in circulating lipoproteins. Alterations in LPL activity may contribute to elevated triglyceride levels observed in patients with this condition [18]. In addition to lipid abnormalities, recent studies indicate that SCH may contribute to cardiovascular risk through other mechanisms, including endothelial dysfunction and increased arterial stiffness [18,19].

Among the various markers used to assess cardiovascular risk, the LDL/HDL ratio is regarded as an important indicator because it reflects the balance between atherogenic lipoproteins and protective lipoproteins [20]. Elevated LDL/HDL ratios are strongly associated with an increased likelihood of developing cardiovascular disease (Kumar, 2020). Patients with SCH frequently exhibit lipid profiles characterized by a higher LDL/HDL ratio, suggesting a more atherogenic lipid pattern and a greater susceptibility to cardiovascular complications [21]. This ratio has been identified as a reliable predictor of cardiovascular risk even in individuals who do not exhibit overt hypothyroidism [22].

Although TSH is widely used as a primary marker for diagnosing thyroid dysfunction, recent evidence suggests that it may also serve as a predictive indicator of lipid abnormalities and cardiovascular risk in patients with SCH. Elevated TSH concentrations have been associated with unfavorable lipid profiles despite normal levels of circulating thyroid hormones, indicating that TSH may reflect underlying metabolic changes related to SCH [23]. Consequently, increasing attention has been directed toward evaluating TSH as a potential independent marker for identifying lipid metabolism disturbances and cardiovascular risk, particularly among individuals without clinically apparent thyroid disease [24]. Recent research has also emphasized the importance of considering TSH levels during the assessment and management of patients with dyslipidemia and cardiovascular risk factors [25,26].

Therefore, the present study aims to investigate the influence of subclinical hypothyroidism on lipid metabolism by examining lipid profile abnormalities, including increased total cholesterol, triglycerides, and LDL-C levels, as well as reduced HDL-C concentrations. In addition, the study evaluates the LDL/HDL ratio as an important indicator of cardiovascular risk in patients with SCH and explores the potential role of TSH as a marker of lipid abnormalities and cardiovascular risk. By comparing lipid profiles and thyroid function parameters between euthyroid individuals and patients diagnosed with subclinical hypothyroidism, this study seeks to determine whether LDL/HDL ratio and TSH levels can serve as reliable indicators of cardiovascular risk and lipid metabolism disturbances in individuals with SCH.

## 2 Patients and Methods

This research utilized a cross-sectional comparative study design to assess variations in lipid metabolism between individuals diagnosed with subclinical hypothyroidism (SCH) and those with normal thyroid function (euthyroid individuals). The investigation was carried out in the clinical laboratory of Novella Polyclinic, where standardized biochemical analysis procedures were applied to maintain consistency and reduce analytical variation.

Ethical approval was granted by the Ethics Committee of the College of Pharmacy, Hawler Medical University (Approval No. HMU-Ec-ph 20240908-145). Prior to participation, all subjects provided verbal informed consent confirming their willingness to be included in the study.

A total of 40 participants were recruited and divided into two groups: 20 individuals with subclinical hypothyroidism and 20 euthyroid controls. Participants were selected from hospital outpatient departments as well as community health screening clinics. The SCH group consisted of individuals with elevated thyroid-stimulating hormone (TSH) levels while maintaining normal free triiodothyronine (FT3) and free thyroxine (FT4) concentrations, confirming a diagnosis of subclinical hypothyroidism. Conversely, the control group included individuals with normal thyroid function tests, including TSH, FT3, and FT4.

To ensure better comparability between the two groups, control participants were matched with SCH patients based on demographic characteristics, including age, sex, and lifestyle habits. This matching approach helped reduce the influence of confounding variables that might affect lipid metabolism.

Clearly defined inclusion and exclusion criteria were implemented to strengthen the study's reliability. Participants were between 12 and 80 years old, covering adolescent and adult populations while avoiding extreme metabolic conditions. Smokers were excluded because smoking is known to influence lipid metabolism and increase cardiovascular risk. In addition, individuals with a family history of thyroid disease were excluded to minimize potential hereditary influences.

Further exclusion criteria included overt thyroid disorders (hyperthyroidism or hypothyroidism), current thyroid hormone replacement therapy, and metabolic conditions such as diabetes mellitus, chronic kidney disease, or liver disease. Pregnant women were also excluded due to hormonal changes that may alter thyroid activity and lipid metabolism. Moreover, participants with a history of cardiovascular disease were not included to prevent bias in the evaluation of lipid profile changes.

The sample size in this study was relatively small due to the strict inclusion and exclusion criteria. It was challenging to identify patients with subclinical hypothyroidism (SCH) who did not present with comorbid conditions, such as cardiovascular disease, and who were not receiving any form of treatment. These restrictions significantly limited the pool of eligible participants.

Furthermore, the age range of participants was relatively broad. This was primarily a consequence of the limited sample size, which restricted the ability to select participants within a narrower and more specific age category.

All participants maintained a normal diet during the study period and reported no engagement in heavy physical exercise or alcohol consumption.

Blood samples were obtained from all participants through standard venipuncture techniques to ensure reliable and comparable measurements. The collected samples were then centrifuged to separate serum from cellular components. The resulting serum was analyzed to measure thyroid function markers (TSH, FT3, and FT4) as well as lipid profile parameters, including total cholesterol, triglycerides, low-density lipoprotein cholesterol (LDL-C), and high-density lipoprotein cholesterol (HDL-C).

All laboratory analyses were conducted using automated biochemical analyzers to ensure precision and reliability. Thyroid hormone levels were measured using the Cobas c411 analyzer, whereas lipid profile parameters were determined using the Cobas c111 analyzer. These instruments provide high sensitivity and accuracy for detecting TSH, FT3, FT4, and lipid markers such as LDL-C, HDL-C, total cholesterol, and triglycerides. Additionally, the LDL/HDL ratio was calculated as an indicator of cardiovascular risk and to further assess lipid abnormalities associated with subclinical hypothyroidism.

Statistical analysis was carried out using SPSS software (version 26.0) along with Python statistical packages, including Pandas, SciPy, and Statsmodels. Descriptive statistics such as mean values and standard deviations were calculated for thyroid function markers (TSH, FT3, FT4) and lipid profile variables (total cholesterol, triglycerides, LDL-C, HDL-C, and LDL/HDL ratio).

To evaluate differences between the SCH group and the euthyroid control group, an independent samples *t*-test was performed. In addition, simple linear regression analysis was conducted to determine whether TSH levels could predict changes in lipid profile parameters, with TSH treated as the independent variable. A value of P < 0.05 was considered statistically significant.

## 3 Result

Table 1 compares thyroid function parameters and lipid profile variables between the subclinical hypothyroid (SCH) group and the euthyroid control group, expressed as mean ± standard deviation (SD), along with the corresponding p-values to determine statistical significance.

**Table 1:** Difference in Mean ± SD of TSH, FT3, FT4, lipid profile and LDL/HDL ratio by study groups.

Parameter	SCH Group (Mean ± SD)	Euthyroid Group (Mean ± SD)	p-value
TSH (IU/L)	9.80 ± 3.99	2.34 ± 1.12	<0.001
FT3 (pmol/L)	4.95 ± 0.80	5.27 ± 0.90	0.1509
FT4 (pmol/L)	13.78 ± 3.92	14.38 ± 2.04	0.4601
Total Cholesterol (mg/dL)	201.8 ± 35.5	189.6 ± 28.4	0.0564
Triglycerides (mg/dL)	165.7 ± 60.8	92.9 ± 39.8	0.0136
LDL (mg/dL)	122.5 ± 40.2	104.3 ± 31.7	0.0564
HDL (mg/dL)	50.8 ± 12.4	58.6 ± 11.3	0.0136
LDL/HDL Ratio	2.25 ± 1.05	1.57 ± 0.56	0.0027

Total cholesterol levels were slightly higher in the SCH group (201.8 ± 35.5 mg/dL) than in the euthyroid group (189.6 ± 28.4 mg/dL), although this difference was not statistically significant (p = 0.0564). Triglyceride levels were significantly elevated in the SCH group (165.7 ± 60.8 mg/dL) compared with the euthyroid group (92.9 ± 39.8 mg/dL), with a statistically significant difference (p = 0.0136).

Low-density lipoprotein (LDL) levels were higher in the SCH group (122.5 ± 40.2 mg/dL) than in the euthyroid group (104.3 ± 31.7 mg/dL), but this difference did not reach statistical significance (p = 0.0564). In contrast, high-density lipoprotein (HDL) levels were significantly lower in the SCH group (50.8 ± 12.4 mg/dL) compared with the euthyroid group (58.6 ± 11.3 mg/dL) (p = 0.0136).

Furthermore, the LDL/HDL ratio was significantly higher in the SCH group (2.25 ± 1.05) than in the euthyroid group (1.57 ± 0.56), indicating a more atherogenic lipid profile among individuals with subclinical hypothyroidism (p = 0.0027).

Overall, these findings suggest that while FT3 and FT4 levels remain comparable, individuals with subclinical hypothyroidism demonstrate significantly elevated TSH levels and notable alterations in lipid metabolism, particularly increased triglycerides, reduced HDL levels, and a higher LDL/HDL ratio, which may contribute to increased cardiovascular risk.

**Fig. 1:**
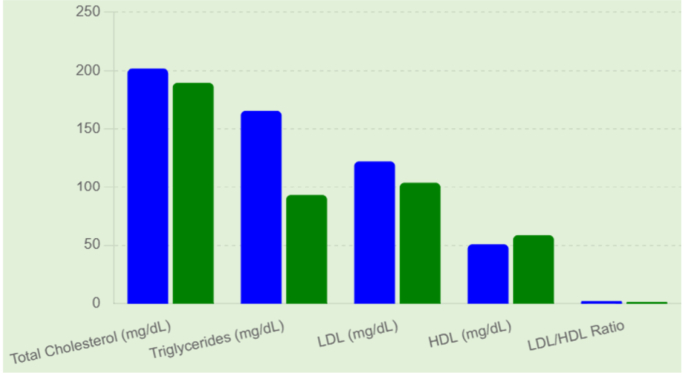
Lipid profile Differences Between SCH and Euthyroid Groups.

Fig. 1 illustrates the comparison of lipid profile parameters between the subclinical hypothyroid (SCH) group and the euthyroid control group. The parameters evaluated include total cholesterol, triglycerides, low-density lipoprotein (LDL), high-density lipoprotein (HDL), and the LDL/HDL ratio.

As shown in the figure, individuals with subclinical hypothyroidism demonstrated higher mean levels of total cholesterol, triglycerides, and LDL compared with the euthyroid group. Triglyceride levels, in particular, showed a noticeable elevation in the SCH group relative to controls. Similarly, LDL concentrations were moderately increased among SCH participants, indicating a tendency toward a more atherogenic lipid profile.

In contrast, HDL levels were lower in the SCH group compared with the euthyroid group. The reduction in HDL, which is considered a protective lipid fraction, further contributes to an unfavorable lipid pattern among individuals with subclinical hypothyroidism.

Consistent with these findings, the LDL/HDL ratio was higher in the SCH group than in the euthyroid group, suggesting an increased cardiovascular risk profile in subjects with subclinical hypothyroidism. Overall, the graphical representation highlights the trend toward dyslipidemia among SCH individuals, characterized by elevated atherogenic lipids and reduced cardioprotective HDL levels.

Table 2 presents the results of the linear regression analysis conducted to evaluate the relationship between thyroid stimulating hormone (TSH) levels and lipid profile parameters, including low-density lipoprotein (LDL), high-density lipoprotein (HDL), triglycerides (TG), total cholesterol (TC), and the LDL/HDL ratio.

**Table 2:** linear regression model with TSH as the dependent (outcome) variable and LDL, HDL, TG, TC and LDL/HDL ratio as explanatory variables.

dependent variables	R-squared (R^2^)	Beta Coefficient	p-Value
LDL	0.06	2.79	0.308
HDL	0.03	−0.56	0.434
Triglycerides	0.05	3.53	0.353
Total Cholesterol	0.007	−0.97	0.892
LDL/HDL Ratio	0.09	0.31	0.178

The regression analysis demonstrated weak associations between TSH levels and the examined lipid parameters. LDL showed a coefficient of determination (R^2^) of 0.06 with a beta coefficient (β) of 2.79; however, this association did not reach statistical significance (p = 0.308). Similarly, HDL exhibited a weak inverse relationship with TSH levels (β = −0.56) and a low explanatory power (R^2^ = 0.03), which was also statistically non-significant (p = 0.434).

Triglycerides showed a modest positive association with TSH levels, with an R^2^ value of 0.05 and a beta coefficient of 3.53, but this relationship did not achieve statistical significance (p = 0.353). Total cholesterol demonstrated a very weak negative association with TSH levels (β = −0.97; R^2^ = 0.007), and this relationship was likewise not statistically significant (p = 0.892).

Among the evaluated parameters, the LDL/HDL ratio exhibited the highest coefficient of determination (R^2^ = 0.09) with a positive beta coefficient (β = 0.31), suggesting a potential trend toward an association with TSH levels. Nevertheless, this relationship also failed to reach statistical significance (p = 0.178).

Overall, the findings of the regression analysis indicate that none of the lipid profile variables were significant predictors of TSH levels within the studied population. Furthermore, the relatively low R^2^ values observed across all variables suggest that lipid parameters explain only a limited proportion of the variability in TSH levels in this cohort.

## 4 Discussion

Subclinical hypothyroidism (SCH) is defined by elevated serum thyroid-stimulating hormone (TSH) concentrations in the presence of normal circulating free thyroxine (FT4). Although often clinically silent, increasing evidence indicates that SCH may influence lipid metabolism and contribute to cardiovascular risk [27,28]. The present study evaluated the relationship between TSH levels and lipid abnormalities in patients with SCH, with particular attention to the LDL/HDL ratio as an indicator of atherogenic risk.

Our findings demonstrate significant alterations in lipid parameters in individuals with SCH compared with euthyroid controls. Specifically, triglyceride levels were significantly higher and HDL-C levels significantly lower in the SCH group. These findings are consistent with previous studies reporting that SCH is associated with atherogenic lipid changes characterized by hypertriglyceridemia and reduced HDL-C concentrations [12,29,30]. Reduced HDL-C levels may impair reverse cholesterol transport, a key protective mechanism that removes excess cholesterol from peripheral tissues and returns it to the liver for excretion. Concurrently, elevated triglyceride concentrations are associated with increased production of triglyceride-rich lipoproteins and the formation of small dense LDL particles, which have greater atherogenic potential [31].

In contrast, total cholesterol and LDL-C levels were only modestly increased in SCH patients and did not reach statistical significance. This observation likely reflects the mild hormonal disturbance characteristic of SCH, where circulating thyroid hormone levels remain within the physiological range despite elevated TSH. In overt hypothyroidism, pronounced reductions in thyroid hormone activity lead to significant hypercholesterolemia primarily due to impaired LDL clearance. In SCH, however, partial preservation of thyroid hormone signaling may attenuate these effects, resulting in more subtle lipid disturbances [28,31].

Despite the relatively modest changes in LDL-C levels, the LDL/HDL ratio was significantly higher in SCH patients compared with euthyroid individuals. The LDL/HDL ratio is widely recognized as a robust predictor of cardiovascular disease and reflects the balance between atherogenic and protective lipoproteins [32,33].

An elevated ratio indicates increased exposure of the vascular endothelium to cholesterol-rich lipoproteins relative to the capacity for reverse cholesterol transport [34,35]. The higher LDL/HDL ratio observed in this study therefore suggests a more atherogenic lipid profile in SCH patients and supports the concept that even mild thyroid dysfunction may contribute to cardiovascular risk.

Regression analysis in our study revealed only weak correlations between TSH levels and lipid parameters. For example, TSH explained approximately 6% of the variability in LDL-C concentrations (R^2^ = 0.06), and the association between TSH and LDL-C was not statistically significant. These findings indicate that TSH alone does not adequately explain lipid abnormalities in SCH and are consistent with several population studies reporting inconsistent associations between TSH and lipid metabolism.

The weak correlation observed in the present study may be explained by the complex regulatory mechanisms governing cholesterol homeostasis. Thyroid hormones influence lipid metabolism through multiple pathways, particularly through regulation of hepatic LDL receptor (LDLR) expression. Triiodothyronine (T3) enhances LDLR transcription via activation of sterol regulatory element-binding protein-2 (SREBP-2), a key transcription factor controlling genes involved in cholesterol uptake and biosynthesis. Increased LDLR expression promotes hepatic clearance of circulating LDL particles, thereby reducing plasma cholesterol levels [36].

In hypothyroid states, decreased thyroid hormone signaling can reduce SREBP-2 activation, leading to decreased LDLR expression and impaired LDL clearance [37]. However, in SCH the presence of normal circulating thyroid hormone concentrations may partially maintain this regulatory pathway, explaining the relatively mild LDL-C changes observed in our cohort.

Another important regulator of LDLR activity is proprotein convertase subtilisin/kexin type 9 (PCSK9), a hepatic protein that promotes lysosomal degradation of LDL receptors. Elevated PCSK9 levels reduce LDLR availability on hepatocyte surfaces and impair LDL clearance. Emerging evidence suggests that thyroid hormone signaling may modulate PCSK9 expression, either directly through transcriptional mechanisms or indirectly through SREBP-2 pathways. Reduced thyroid hormone activity may therefore increase PCSK9 expression, further limiting LDL receptor recycling and contributing to dyslipidemia [[37], [38], [39]].

While traditional models attribute lipid abnormalities in hypothyroidism primarily to reduced thyroid hormone action, recent studies have proposed that TSH itself may exert direct metabolic effects. Hepatic cells and adipocytes have been shown to express TSH receptors, raising the possibility that elevated TSH may influence lipid metabolism independently of thyroid hormones [40]. Experimental studies suggest that TSH signaling may stimulate hepatic cholesterol synthesis and potentially increase PCSK9 expression. However, clinical evidence remains inconsistent, and many population-based studies have failed to demonstrate strong correlations between TSH concentrations and lipid parameters [36,37]. The weak associations observed in our study support the interpretation that TSH is more likely a marker of thyroid axis dysregulation rather than a primary determinant of lipid abnormalities in SCH.

Multiple additional factors may also contribute to lipid disturbances in SCH, including altered lipoprotein lipase activity, insulin resistance, genetic predisposition, dietary patterns, and body composition. These factors may interact with mild thyroid dysfunction to produce heterogeneous metabolic phenotypes, further explaining the variability reported in previous studies [31,41,42].

The present findings have important translational implications. A better understanding of thyroid-regulated lipid metabolism may inform the development of targeted therapies for dyslipidemia and cardiovascular disease. One promising therapeutic strategy involves inhibition of PCSK9. Monoclonal antibodies targeting PCSK9 markedly increase LDL receptor recycling and enhance hepatic LDL clearance, resulting in substantial reductions in circulating LDL-C levels [38]. If thyroid dysfunction contributes to increased PCSK9 activity, PCSK9 inhibitors may represent a particularly effective treatment strategy for patients with dyslipidemia associated with SCH.

Another emerging approach involves selective activation of hepatic thyroid hormone receptor-β (THR-β). THR-β agonists mimic the lipid-lowering effects of thyroid hormones while minimizing systemic adverse effects such as tachycardia and bone loss [43]. Resmetirom, a selective THR-β agonist, has demonstrated the ability to improve lipid profiles and reduce hepatic steatosis in clinical trials. By enhancing LDL receptor expression and stimulating hepatic lipid metabolism, THR-β agonists may offer a novel therapeutic approach for metabolic disorders associated with impaired thyroid hormone signaling [44].

Future research should further investigate the molecular mechanisms linking thyroid function and lipid metabolism, particularly the regulatory interactions among SREBP-2, PCSK9, and LDLR pathways. Experimental studies using molecular techniques such as quantitative PCR and Western blot analysis could evaluate expression levels of LDLR, PCSK9, and SREBP-2 in response to changes in thyroid hormone signaling. Measurement of circulating PCSK9 concentrations using enzyme-linked immunosorbent assays may also provide insight into the contribution of this pathway to lipid abnormalities in SCH. Additionally, luciferase reporter assays incorporating thyroid hormone response elements could help elucidate transcriptional regulation mediated by thyroid hormone receptors.

Importantly, lipid metabolism is influenced by a wide range of non-thyroidal factors. Recent high-quality systematic reviews and meta-analyses have demonstrated that nutritional and dietary interventions significantly modulate lipid profiles and cardiovascular risk markers. For example, vitamin D supplementation has been associated with modest improvements in lipid parameters, although findings remain heterogeneous across populations [45]. Similarly, phytosterols have consistently been shown to reduce LDL-C levels by inhibiting intestinal cholesterol absorption. Dietary fibers such as guar gum have demonstrated lipid-lowering effects, particularly in reducing total cholesterol and LDL-C [46].

In addition, dietary patterns such as the ketogenic diet have been extensively studied, with meta-analyses indicating complex effects on lipid metabolism, including reductions in triglycerides but variable impacts on LDL-C [47]. Capsaicin-based interventions have also been reported to exert favorable metabolic effects, potentially through enhanced lipid oxidation and improved endothelial function. Collectively, these findings underscore that dyslipidemia is a multifactorial condition shaped by interactions between hormonal regulation, dietary intake, metabolic status, and genetic predisposition [48].

The integration of these findings provides important context for interpreting lipid abnormalities in SCH. It is likely that mild thyroid dysfunction interacts with modifiable lifestyle factors to produce heterogeneous lipid phenotypes, which may explain the variability observed across studies and the weak correlation between TSH and lipid parameters.

Several limitations should be considered when interpreting the results of this study. The cross-sectional design precludes causal inference, and lipid abnormalities may be influenced by confounding factors such as diet, insulin resistance, and genetic predisposition. Furthermore, the absence of direct measurements of PCSK9 or LDL receptor activity limits mechanistic interpretation. Nevertheless, the findings provide valuable insight into the relationship between SCH and lipid metabolism and highlight the importance of cardiovascular risk assessment in patients with mild thyroid dysfunction.

## 5 Conclusion

Patients with subclinical hypothyroidism exhibit significant alterations in lipid metabolism characterized by elevated triglycerides, reduced HDL-C, and an increased LDL/HDL ratio. Although TSH levels were markedly elevated in SCH patients, their correlation with lipid parameters was weak, suggesting that lipid abnormalities arise from complex metabolic mechanisms rather than direct TSH effects alone. These findings underscore the importance of comprehensive lipid monitoring in SCH patients and support further investigation into molecular pathways linking thyroid function to cholesterol metabolism.

Further longitudinal and interventional studies are needed to determine whether thyroid hormone replacement therapy, particularly levothyroxine treatment, can improve lipid profiles and reduce long-term cardiovascular risk in patients with SCH. While several studies have suggested potential benefits of thyroid hormone therapy in selected patients, the optimal treatment thresholds and patient populations that may derive the greatest cardiovascular benefit remain subjects of ongoing investigation.

Future clinical trials should therefore evaluate the effects of thyroid hormone replacement on lipid metabolism, LDL receptor activity, and cardiovascular outcomes in SCH populations. Such studies may help clarify whether early therapeutic intervention could serve as a preventive strategy against atherosclerosis and cardiovascular disease in patients with mild thyroid dysfunction.

In conclusion, the present findings support the growing recognition that subclinical hypothyroidism is associated with subtle but clinically relevant lipid disturbances. Continued research integrating clinical observations with molecular and therapeutic investigations will be essential to better understand the cardiovascular implications of SCH and to develop effective strategies for risk reduction and patient management.

## 6 Recommendations

Based on the findings of this study, routine evaluation of lipid profiles should be considered in patients diagnosed with subclinical hypothyroidism, even in the absence of overt clinical symptoms. Particular attention should be given to the LDL/HDL ratio, which may serve as a sensitive indicator of early cardiovascular risk in this population. Early identification of dyslipidemia may allow clinicians to implement preventive strategies such as lifestyle modification, dietary counseling, and appropriate lipid-lowering therapy when necessary.

Furthermore, clinicians should carefully monitor thyroid function and cardiovascular risk factors in patients with SCH, especially in those with additional metabolic conditions such as obesity, insulin resistance, or diabetes mellitus. Although current clinical guidelines remain cautious regarding routine treatment of SCH, thyroid hormone replacement therapy may be considered in selected patients with persistent TSH elevation and unfavorable lipid profiles. Future clinical studies will be important in determining whether early therapeutic intervention can improve lipid metabolism and reduce long-term cardiovascular morbidity in this population.

## Declaration of competing interest

I am Muhanad Salah Mawlood, hereby declare that I have no personal or financial interests that may be perceived as influencing the impartiality of my work in relation to my research The Unseen Impact of Subclinical Hypothyroidism on Lipid Profile and Cardiovascular Risk.

## Data Availability

The datasets generated and analyzed during the current study are not publicly available due to privacy and ethical restrictions but are available from the corresponding author on reasonable request. All data supporting the findings of this study were obtained from laboratory analyses conducted at Novella Polyclinic and were used solely for the purposes of this research in accordance with ethical approval from the Ethics Committee of the College of Pharmacy, Hawler Medical University.

## References

[1] Rodondi N., Newman A.B., Vittinghoff E., de Rekeneire N., Satterfield S., Harris T.B. (2005 Nov 28). Subclinical hypothyroidism and the risk of heart failure, other cardiovascular events, and death. Arch. Intern. Med..

[2] Hadi V., Pahlavani N., Malekahmadi M., Nattagh-Eshtivani E., Navashenaq J.G., Hadi S. (2021 Jan 1). *Nigella sativa* in controlling Type 2 diabetes, cardiovascular, and rheumatoid arthritis diseases: molecular aspects. J. Res. Med. Sci..

[3] Peeters R.P. (2017 Jun 29). Subclinical hypothyroidism. N. Engl. J. Med..

[4] (2013). 2013 ETA guideline: management of subclinical hypothyroidism. Eur. Thyroid J..

[5] Pearce E.N. (2004 Nov). Hypothyroidism and dyslipidemia: modern concepts and approaches. Curr. Cardiol. Rep..

[6] Sabatino L., Vassalle C. (2025 Mar). Thyroid hormones and metabolism regulation: which role on brown adipose tissue and browning process?. Biomolecules.

[7] Biological and pharmacological effects and nutritional impact of phytosterols: a comprehensive review [Internet]. [cited 2026 March 13]. Available from: https://onlinelibrary.wiley.com/doi/epdf/10.1002/ptr.7312?msockid=2b4a35559ba16f6b3abe24979a7e6ea5. 10.1002/ptr.7312 34729825

[8] Guan Y., Liu X., Yang Z., Zhu X., Liu M., Du M. (2025 May 27). PCSK9 promotes LDLR degradation by preventing SNX17-Mediated LDLR recycling. Circulation.

[9] Grejtakova D., Boronova I., Bernasovska J., Bellosta S. (2025 Dec). PCSK9 and lipid metabolism: genetic variants, current therapies, and cardiovascular outcomes. Cardiovasc. Drugs Ther..

[10] Mahjoubin-Tehran M., Rezaei S., Santos R.D., Jamialahmadi T., Almahmeed W., Sahebkar A. (2024 May 25). Targeting PCSK9 as a key player in lipid metabolism: exploiting the therapeutic and biosensing potential of aptamers. Lipids Health Dis..

[11] Ajoolabady A., Pratico D., Mazidi M., Davies I.G., Lip G.Y.H., Seidah N. (2025 Feb). PCSK9 in metabolism and diseases. Metabolism.

[12] Duntas L.H. (2002 Apr). Thyroid disease and lipids. Thyroid®.

[13] Aljabri K., Alnasser M., Facharatz Bokhari S., Alshareef M., Khan P. (2019 Jan 1). The frequency of hypothyroidism in Saudi community-based hospital: a retrospective single centre study. Trends Diabetes Metabol..

[14] Subclinical hypothyroidism in older individuals - the lancet diabetes & endocrinology [internet]. [cited 2026 March 13]. Available from: https://www.thelancet.com/journals/landia/article/PIIS2213-8587(21)00285-0/abstract. 10.1016/S2213-8587(21)00285-0 34953533

[15] Biondi B., Cappola A.R., Cooper D.S. (2019 Jul 9). Subclinical hypothyroidism: a review. JAMA.

[16] Patrizio A., Ferrari S.M., Elia G., Ragusa F., Balestri E., Botrini C. (2024 Jun). Hypothyroidism and metabolic cardiovascular disease. Front. Endocrinol..

[17] Kumar A., Prabhu M., Bhat N., Stanley W. (2022 Sep 12). A study of cardiovascular profile in patients with primary hypothyroidism. Biomedicine.

[18] Liu F.H., Hwang J.S., Kuo C.F., Ko Y.S., Chen S.T., Lin J.D. (2018 Feb 1). Subclinical hypothyroidism and metabolic risk factors association: a health examination-based study in northern Taiwan. Biomed. J..

[19] Grais I.M., Sowers J.R. (2014 Aug). Thyroid and the heart. Am. J. Med..

[20] Tagami T., Kimura H., Ohtani S., Tanaka T., Tanaka T., Hata S. (2011). Multi-center study on the prevalence of hypothyroidism in patients with hypercholesterolemia. Endocr. J..

[21] Rizos C.V., Elisaf M.S., Liberopoulos E.N. (2011). Effects of thyroid dysfunction on lipid profile. Open Cardiovasc. Med. J..

[22] Pertseva N., Einer X. (2021 Mar 31). Metabolic changes of lipids in patient with subclinical hypothyroidism. Romanian J. Diabet. Nutr. Metabol. Dis..

[23] Xiao C., Dash S., Morgantini C., Hegele R.A., Lewis G.F. (2016 Jul). Pharmacological targeting of the atherogenic dyslipidemia complex: the next frontier in CVD prevention beyond lowering LDL cholesterol. Diabetes.

[24] Kottagi S., Rathi D., Dongre N. (2014 Jan 1).

[25] Neves C., Alves M., Medina J.L., Delgado J.L. (2008 Oct). Thyroid diseases, dyslipidemia and cardiovascular pathology. Rev. Port. Cardiol..

[26] Kalantari S., Naghipour M. (2014 Aug 19). Statin therapy and hepatotoxicity: appraisal of the safety profile of atorvastatin in hyperlipidemic patients. Adv. Biomed. Res..

[27] Wang X., Wu Z., Liu Y., Wu C., Jiang J., Hashimoto K. (2024 Oct 15). The role of thyroid-stimulating hormone in regulating lipid metabolism: implications for body–brain communication. Neurobiol. Dis..

[28] Chiovato L., Magri F., Carlé A. (2019 Sep). Hypothyroidism in context: where we’ve been and where we’re going. Adv. Ther..

[29] Pearce S.H.S., Brabant G., Duntas L.H., Monzani F., Peeters R.P., Razvi S. (2013 Dec). 2013 ETA guideline: management of subclinical hypothyroidism. Eur. Thyroid J..

[30] (2025 May 1). Is subclinical hypothyroidism associated with cardiovascular disease in the elderly?. Endocrine Connections.

[31] Nicolaou M., Toumba M. (2024 Dec). Lipid profile pitfalls in subclinical hypothyroidism pathophysiology and treatment. Lipidology.

[32] Iglesias P., Díez J.J. (2007 Nov). Influence of thyroid dysfunction on serum concentrations of adipocytokines. Cytokine.

[33] Sun T., Chen M., Shen H., PingYin null, Fan L., Chen X. (2022 Jun 17). Predictive value of LDL/HDL ratio in coronary atherosclerotic heart disease. BMC Cardiovasc. Disord..

[34] Luo Y., Wu F., Huang Z., Gong Y., Zheng Y. (2022 Dec 13). Assessment of the relationship between subclinical hypothyroidism and blood lipid profile: reliable or not?. Lipids Health Dis..

[35] Duntas L., Chiovato L. (2014 Aug). Cardiovascular risk in patients with subclinical hypothyroidism. Eur. Endocrinol..

[36] Pedrelli M., Pramfalk C., Parini P. (2010 Dec 21). Thyroid hormones and thyroid hormone receptors: effects of thyromimetics on reverse cholesterol transport. World J. Gastroenterol..

[37] Ozkan-Nikitaras T., Grzesik D.J., Romano L.E.L., Chapple J.P., King P.J., Shoulders C.C. (2024 Jan). N-SREBP2 provides a mechanism for dynamic control of cellular cholesterol homeostasis. Cells.

[38] Nair T. (2024 Mar). Role of PCSK9 inhibitors in the management of dyslipidaemia. Indian Heart J..

[39] Bonde Y., Breuer O., Lütjohann D., Sjöberg S., Angelin B., Rudling M. (2014 Nov 1). Thyroid hormone reduces PCSK9 and stimulates bile acid synthesis in humans[S]. JLR (J. Lipid Res.).

[40] Zhang W., Tian L min, Han Y., yan Ma H., cheng Wang L., Guo J. (2009). Presence of thyrotropin receptor in hepatocytes: not a case of illegitimate transcription. J. Cell Mol. Med..

[41] Zhang D., Wei Y., Huang Q., Chen Y., Zeng K., Yang W. (2022 Oct 19). Important hormones regulating lipid metabolism. Molecules.

[42] Wyne K.L., Nair L., Schneiderman C.P., Pinsky B., Antunez Flores O., Guo D. (2022 Nov 10). Hypothyroidism prevalence in the United States: a retrospective study combining national health and nutrition examination survey and claims data, 2009–2019. J. Endocr. Soc..

[43] Saponaro F., Sestito S., Runfola M., Rapposelli S., Chiellini G. (2020 Jul 9). Selective thyroid hormone receptor-beta (TRβ) agonists: new perspectives for the treatment of metabolic and neurodegenerative disorders. Front. Med..

[44] (2025). Resmetirom: a promising treatment for non-alcoholic steatohepatitis. Am. J. Pharmacother. Pharmaceut. Sci..

[45] Hu L., Velu P., Prabahar K., Hernández-Wolters B., Kord-Varkaneh H., Xu Y. (2025 Sep 1). Effect of vitamin D supplementation on lipid profile in overweight or Obese women: a meta-analysis and systematic review of randomized controlled trials. Nutr. Rev..

[46] Xia W., Xiang S., Gaman M.A., Jamilian P., Prabahar K., Du G. (2022 Dec). The effects of phytosterol and phytostanol supplementation on the lipid profile in postmenopausal women: a systematic review and meta-analysis of randomized controlled trials. Phytother Res..

[47] Daley S.F., Masood W., Annamaraju P., Khan Suheb M.Z. (2026). StatPearls [Internet].

[48] Yang Q., La C., Prabahar K., Safargar M., Kord-Varkaneh H., null Temuqile (2025 Nov). The effect of capsaicin, capsinoids, and pepper-based interventions on lipid profiles in overweight or obese individuals: a systematic review and meta-analysis of randomized controlled trials. Diabetes Res. Clin. Pract..

