# Supplementary material for: The unseen impact of subclinical hypothyroidism on lipid profile and cardiovascular risk
Source: Biochem Biophys Rep. 2026 Apr 24;46:102605. doi: 10.1016/j.bbrep.2026.102605 (PMC13127173; doi:10.1016/j.bbrep.2026.102605)
Supplement: Multimedia component 1 [file mmc1.docx]

STROBE Statement—Checklist of items that should be included in reports of ***cross-sectional studies***

|  | Item No | Recommendation | Page No |
| --- | --- | --- | --- |
| **Title and abstract** | 1 | Th Unseen Impact of Subclinical Hypothyroidism on Lipid Profile and Cardiovascular Risk | I |
|  |  | **Background:** Subclinical hypothyroidism (SCH), defined by elevated thyroid-stimulating hormone (TSH) with normal circulating thyroid hormones, is a common endocrine disorder that frequently remains clinically silent. Emerging evidence suggests that even mild thyroid dysfunction may influence lipid metabolism and contribute to early cardiovascular risk. However, the extent to which TSH levels reflect lipid abnormalities in SCH remains controversial.  **Objective:** This study aimed to evaluate the influence of SCH on lipid metabolism and to determine whether TSH levels are associated with alterations in lipid profile parameters and atherogenic cardiovascular risk markers.  **Methods:** A cross-sectional comparative study was conducted including 40 participants: 20 patients diagnosed with SCH and 20 euthyroid controls matched for demographic characteristics. Serum concentrations of TSH, free triiodothyronine (FT3), and free thyroxine (FT4) were measured alongside lipid profile parameters, including total cholesterol, triglycerides, low-density lipoprotein cholesterol (LDL-C), and high-density lipoprotein cholesterol (HDL-C). The LDL/HDL ratio was calculated as an indicator of atherogenic cardiovascular risk. Independent sample *t*-tests were applied to compare study groups, and linear regression analysis was performed to evaluate associations between TSH levels and lipid parameters.  **Results:** Individuals with SCH demonstrated significant lipid alterations compared with euthyroid controls. Triglyceride levels were significantly higher (165.7 ± 60.8 vs. 92.9 ± 39.8 mg/dL; *p* = 0.0136), while HDL-C levels were significantly lower (50.8 ± 12.4 vs. 58.6 ± 11.3 mg/dL; *p* = 0.0136). The LDL/HDL ratio was also significantly elevated in the SCH group (2.25 ± 1.05 vs. 1.57 ± 0.56; *p* = 0.0027), indicating a more atherogenic lipid profile. In contrast, total cholesterol and LDL-C showed modest but statistically non-significant increases. Regression analysis revealed weak and non-significant correlations between TSH levels and lipid parameters.  **Conclusion:** SCH is associated with unfavorable lipid alterations characterized by elevated triglycerides, reduced HDL-C, and an increased LDL/HDL ratio, suggesting early atherogenic risk despite normal thyroid hormone levels. These findings highlight the importance of comprehensive lipid assessment in SCH and suggest that the LDL/HDL ratio may serve as a more sensitive indicator of cardiovascular risk than TSH alone. | II-III |
| Introduction | | | |
| Background/rationale | 2 | SCH is defined by increased serum TSH concentrations while circulating levels of T3 and T4 remain within the normal range. This condition is frequently asymptomatic and often goes undetected unless identified through routine laboratory investigations. Although individuals with SCH generally do not present with typical symptoms of hypothyroidism—such as fatigue, weight gain, and cold intolerance—accumulating evidence suggests that even this subtle thyroid dysfunction may produce metabolic effects, particularly in relation to lipid metabolism. | 4 |
| Objectives | 3 | the present study aims to investigate the influence of subclinical hypothyroidism on lipid metabolism by examining lipid profile abnormalities, including increased total cholesterol, triglycerides, and LDL-C levels, as well as reduced HDL-C concentrations. In addition, the study evaluates the LDL/HDL ratio as an important indicator of cardiovascular risk in patients with SCH and explores the potential role of TSH as a marker of lipid abnormalities and cardiovascular risk. By comparing lipid profiles and thyroid function parameters between euthyroid individuals and patients diagnosed with subclinical hypothyroidism, this study seeks to determine whether LDL/HDL ratio and TSH levels can serve as reliable indicators of cardiovascular risk and lipid metabolism disturbances in individuals with SCH. | 6 |
| Methods | | | |
| Study design | 4 | This research utilized a cross-sectional comparative study design to assess variations in lipid metabolism between individuals diagnosed with subclinical hypothyroidism (SCH) and those with normal thyroid function (euthyroid individuals). The investigation was carried out in the clinical laboratory of Novella Polyclinic, where standardized biochemical analysis procedures were applied to maintain consistency and reduce analytical variation. | 7 |
| Setting | 5 | A total of 40 participants were recruited and divided into two groups: 20 individuals with subclinical hypothyroidism and 20 euthyroid controls. Participants were selected from hospital outpatient departments as well as community health screening clinics. The SCH group consisted of individuals with elevated thyroid-stimulating hormone (TSH) levels while maintaining normal free triiodothyronine (FT3) and free thyroxine (FT4) concentrations, confirming a diagnosis of subclinical hypothyroidism. Conversely, the control group included individuals with normal thyroid function tests, including TSH, FT3, and FT4. | 7 |
| Participants | 6 | A total of 40 participants were recruited and divided into two groups: 20 individuals with subclinical hypothyroidism and 20 euthyroid controls. Participants were selected from hospital outpatient departments as well as community health screening clinics. | 7 |
|  |  |  |  |
| Data sources/ measurement | 7 | Blood samples were obtained from all participants through standard venipuncture techniques to ensure reliable and comparable measurements. The collected samples were then centrifuged to separate serum from cellular components. The resulting serum was analyzed to measure thyroid function markers (TSH, FT3, and FT4) as well as lipid profile parameters, including total cholesterol, triglycerides, low-density lipoprotein cholesterol (LDL-C), and high-density lipoprotein cholesterol (HDL-C). | **8** |
|  |  |  |  |
| Study size | 8 | A total of 40 participants were recruited and divided into two groups: 20 individuals with subclinical hypothyroidism and 20 euthyroid controls | 7 |
| Quantitative variables | 9 | All laboratory analyses were conducted using automated biochemical analyzers to ensure precision and reliability. Thyroid hormone levels were measured using the Cobas c411 analyzer, whereas lipid profile parameters were determined using the Cobas c111 analyzer. These instruments provide high sensitivity and accuracy for detecting TSH, FT3, FT4, and lipid markers such as LDL-C, HDL-C, total cholesterol, and triglycerides. Additionally, the LDL/HDL ratio was calculated as an indicator of cardiovascular risk and to further assess lipid abnormalities associated with subclinical hypothyroidism. | 8 |
| Statistical methods | 10 | (*a*) Statistical analysis was carried out using SPSS software (version 26.0) along with Python statistical packages, including Pandas, SciPy, and Statsmodels | 9 |
|  |  | (*b*) Descriptive statistics such as mean values and standard deviations were calculated for thyroid function markers (TSH, FT3, FT4) and lipid profile variables (total cholesterol, triglycerides, LDL-C, HDL-C, and LDL/HDL ratio). | 9 |
|  |  |  |  |
|  |  | To evaluate differences between the SCH group and the euthyroid control group, an independent samples t-test was performed. In addition, simple linear regression analysis was conducted to determine whether TSH levels could predict changes in lipid profile parameters, with TSH treated as the independent variable. A value of P< 0.05 was considered statistically significant. | 9 |
|  |  |  |  |
| Results | | | |
|  | 11 | (a) Table Table 1 compares thyroid function parameters and lipid profile variables between the subclinical hypothyroid (SCH) group and the euthyroid control group, expressed as mean ± standard deviation (SD), along with the corresponding p-values to determine statistical significance.  Total cholesterol levels were slightly higher in the SCH group (201.8 ± 35.5 mg/dL) than in the euthyroid group (189.6 ± 28.4 mg/dL), although this difference was not statistically significant (p = 0.0564). Triglyceride levels were significantly elevated in the SCH group (165.7 ± 60.8 mg/dL) compared with the euthyroid group (92.9 ± 39.8 mg/dL), with a statistically significant difference (p = 0.0136).  Low-density lipoprotein (LDL) levels were higher in the SCH group (122.5 ± 40.2 mg/dL) than in the euthyroid group (104.3 ± 31.7 mg/dL), but this difference did not reach statistical significance (p = 0.0564). In contrast, high-density lipoprotein (HDL) levels were significantly lower in the SCH group (50.8 ± 12.4 mg/dL) compared with the euthyroid group (58.6 ± 11.3 mg/dL) (p = 0.0136).  Furthermore, the LDL/HDL ratio was significantly higher in the SCH group (2.25 ± 1.05) than in the euthyroid group (1.57 ± 0.56), indicating a more atherogenic lipid profile among individuals with subclinical hypothyroidism (p = 0.0027).  Overall, these findings suggest that while FT3 and FT4 levels remain comparable, individuals with subclinical hypothyroidism demonstrate significantly elevated TSH levels and notable alterations in lipid metabolism, particularly increased triglycerides, reduced HDL levels, and a higher LDL/HDL ratio, which may contribute to increased cardiovascular risk. | 10 |
|  |  |  |  |
|  |  |  |  |
|  |  |  |  |
|  |  |  |  |
|  |  |  |  |
|  | 12 | (b) Table 2 presents the results of the linear regression analysis conducted to evaluate the relationship between thyroid stimulating hormone (TSH) levels and lipid profile parameters, including low-density lipoprotein (LDL), high-density lipoprotein (HDL), triglycerides (TG), total cholesterol (TC), and the LDL/HDL ratio.  The regression analysis demonstrated weak associations between TSH levels and the examined lipid parameters. LDL showed a coefficient of determination (R²) of 0.06 with a beta coefficient (β) of 2.79; however, this association did not reach statistical significance (p = 0.308). Similarly, HDL exhibited a weak inverse relationship with TSH levels (β = −0.56) and a low explanatory power (R² = 0.03), which was also statistically non-significant (p = 0.434).  Triglycerides showed a modest positive association with TSH levels, with an R² value of 0.05 and a beta coefficient of 3.53, but this relationship did not achieve statistical significance (p = 0.353). Total cholesterol demonstrated a very weak negative association with TSH levels (β = −0.97; R² = 0.007), and this relationship was likewise not statistically significant (p = 0.892).  Among the evaluated parameters, the LDL/HDL ratio exhibited the highest coefficient of determination (R² = 0.09) with a positive beta coefficient (β = 0.31), suggesting a potential trend toward an association with TSH levels. Nevertheless, this relationship also failed to reach statistical significance (p = 0.178).  Overall, the findings of the regression analysis indicate that none of the lipid profile variables were significant predictors of TSH levels within the studied population. Furthermore, the relatively low R² values observed across all variables suggest that lipid parameters explain only a limited proportion of the variability in TSH levels in this cohort. | 12-13 |
|  |  |  |  |
|  |  |  |  |
|  |  |  |  |
| Discussion | | | |
| Key results | 13 | Individuals with SCH demonstrated significant lipid alterations compared with euthyroid controls. Triglyceride levels were significantly higher (165.7 ± 60.8 vs. 92.9 ± 39.8 mg/dL; *p* = 0.0136), while HDL-C levels were significantly lower (50.8 ± 12.4 vs. 58.6 ± 11.3 mg/dL; *p* = 0.0136). The LDL/HDL ratio was also significantly elevated in the SCH group (2.25 ± 1.05 vs. 1.57 ± 0.56; *p* = 0.0027), indicating a more atherogenic lipid profile. In contrast, total cholesterol and LDL-C showed modest but statistically non-significant increases. Regression analysis revealed weak and non-significant correlations between TSH levels and lipid parameters. | 2 |
| Limitations | 14 | .None of the lipid profile variables were significant predictors of TSH levels within the studied population.. | 11 |
| Interpretation | 15 | Further longitudinal and interventional studies are needed to determine whether thyroid hormone replacement therapy, particularly levothyroxine treatment, can improve lipid profiles and reduce long-term cardiovascular risk in patients with SCH. While several studies have suggested potential benefits of thyroid hormone therapy in selected patients, the optimal treatment thresholds and patient populations that may derive the greatest cardiovascular benefit remain subjects of ongoing investigation.  Future clinical trials should therefore evaluate the effects of thyroid hormone replacement on lipid metabolism, LDL receptor activity, and cardiovascular outcomes in SCH populations. Such studies may help clarify whether early therapeutic intervention could serve as a preventive strategy against atherosclerosis and cardiovascular disease in patients with mild thyroid dysfunction. | 18 |
|  |  |  |  |
| Other information | | | |
| Funding | 16 | This research is not funded by any organization, it's funded solely by the author |  |
